# Selection of cell-type specific antibodies on tissue-sections using phage display

**DOI:** 10.1111/jcmm.12568

**Published:** 2015-03-26

**Authors:** Simon Asbjørn Larsen, Theresa Meldgaard, Simon Lykkemark, Ole Aalund Mandrup, Peter Kristensen

**Affiliations:** a Department of Molecular Biology and Genetics, Aarhus UniversityAarhus, Denmark; b Department of Engineering, Aarhus UniversityAarhus, Denmark; c Department of Clinical Medicine, Aarhus UniversityAarhus, Denmark; d Sino-Danish Centre for Education and Research (SDC)Aarhus, Denmark

**Keywords:** phage display, biomarkers, recombinant antibody selection, tissue, vascular targeting, endothelial cells, rare cells

## Abstract

With the advent of modern technologies enabling single cell analysis, it has become clear that small sub-populations of cells or even single cells can drive the phenotypic appearance of tissue, both diseased and normal. Nucleic acid based technologies allowing single cell analysis has been faster to mature, while technologies aimed at analysing the proteome at a single cell level is still lacking behind, especially technologies which allow single cell analysis in tissue. Introducing methods, that allows such analysis, will pave the way for discovering new biomarkers with more clinical relevance, as these may be unique for microenvironments only present in tissue and will avoid artifacts introduced by *in vitro* studies. Here, we introduce a technology enabling biomarker identification on small sub-populations of cells within a tissue section. Phage antibody libraries are applied to the tissue sections, followed by washing to remove non-bound phage particles. To eliminate phage antibodies binding to antigens ubiquitously expressed and retrieve phage antibodies binding specifically to antigens expressed by the sub-population of cells, the area of interest is protected by a ‘shadow stick’. The phage antibodies on the remaining areas on the slide are exposed to UV light, which introduces cross-links in the phage genome, thus rendering them non-replicable. In this work we applied the technology, guided by CD31 expressing endothelial cells, to isolate recombinant antibodies specifically binding biomarkers expressed either by the cell or in the microenvironment surrounding the endothelial cell.

## Introduction

Great advancements have been made in understanding the molecular mechanisms of the endothelial and perivascular cells composing the blood vessel. These cells play complex roles in both the healthy blood vasculature system and in cardiovascular disease. The interplay between the endothelial and perivascular cells regulates the blood flow and facilitates the transport of gases, solutes and cells. In disease, endothelial dysfunction especially challenges the vasomotion capability controlled by the vascular smooth muscle cells, which may lead to the onset of atherosclerosis and cardiovascular disease 1,2. Impairment of the endothelial function is also associated with hypertension and hypercholesterolaemia 3,4. The inner lining of the blood vasculature is thus a major site for several pathological processes that contribute to cardiovascular disease. Recently, studies have put focus on the important supportive role of the perivascular cells 5. The perivascular cells communicate with the endothelial cells, either by direct physical contact and gap junctions or by paracrine signalling pathways. Examples of perivascular cells are the smooth muscle cells and the pericytes. Furthermore, neovascularization and angiogenesis are implicated in a wide variety of pathological events, such as wound repair, inflammatory disorders, diabetic retinopathy and tumour angiogenesis 6. A great diversity of cells are involved in these processes, such as endothelial- and pericyte progenitor cells, hematopoietic stem cells and mesenchymal stem cells 7.

The inherent heterogeneity of the cells composing the blood vessels provides diverse and specialized functions at different anatomical sites. Detailed knowledge of the involved cells and their cellular crosstalk is crucial for the development of clinical therapies for vascular diseases and tumour angiogenesis. However, this process is complicated by the fact that some of these cells are rare or exists in different maturation stages 8. Moreover, they are difficult to identify, isolate and enrich due to the lack of specific biomarkers.

Phage display is a widely used method to generate cell-specific recombinant antibody fragments which may be utilized in research, diagnostics or clinical treatment 9,10. Following the trend of developing targeted therapeutics, the pharmaceutical and biotechnology industry are increasing their focus on monoclonal antibodies 11,12. In phage display, the sequence coding for the antibody fragment is fused with the gene encoding one of the viral coat proteins. This fusion causes the antibody fragment to be displayed on the surface of the phage particle where it is free to interact with any target. Hereby, the phage particle connects the phenotype of the antibody fragment with its genotype 13. Today, many libraries have been developed with different recombinant antibody fragments 14,15. Phage libraries are most often in the form of either fragment antigen binding, single chain fragment variable (scFv) or single domain antibodies.

The discovery of cell-specific antibody fragments and identification of the corresponding antigen may unearth novel biomarkers. However, it is complicated to perform antibody fragment selection against single cells mixed within a heterogeneous population. Selection performed on whole cell populations would preferentially target the highly expressed and common antigens. Unique or upregulated biomarkers expressed on the single cells of interest may not be identified unless the selection procedure is restricted to only these cells. Previous published work performed in our laboratory, describes a method to select specific antibody fragments against a single rare cell residing in a heterogeneous population using a novel shadow stick technology 16–18. This technique allows for antibody fragment selection against a rare cell that can be separated into suspension. Such rare cells may originate from circulation in the peripheral blood, culture or from solubilized tissue.

In the present study, we describe a method to select antibody fragments by phage display directly on tissue sections. In tissue, the target cells are embedded within their natural environment. The neighbouring cells and extracellular matrix form a unique surrounding niche 19–21. The interplay between the target cells and their surroundings influences their behaviour and hence play an important role in the antigens expressed 22,23. One relevant example of this interaction is the dynamic regulation of cellular adhesion molecules in the microenvironment. These molecules respond to tissue alterations and are among other things involved in the naturally occurring mechanism ‘angiogenic switch’ 24,25, which is also involved in pathological conditions 26. Cultured or solubilized cells stripped from their niche may change in numerous ways with respect to expression of potentially unique antigens. Cultured cells may down-regulate expression of molecules, which only serve a specific function in context of the natural surroundings. In addition to proteolytic destruction during solubilization unique and/or rare epitopes may lose their biologically active conformation, rendering selected antibody fragments inapplicable 27. Hence, compared to cultured or solubilized cells tissue provides a potential source of unique and otherwise inaccessible antigens, while optimally preserving the epitopes from unauthentic manipulation.

A further advantage provided by tissue, is the opportunity to target cells based on their morphology. If certain cells in a tissue sample are deemed interesting by any relevant criteria, the shadow stick technique allows for straight forward selection. Previously described selection methods on solubilized or cultured cells requires several preluding steps before selection, including solubilizing the tissue, and a subsequent sorting of the target cells 28,29. However, these steps prove troublesome to perform without the means to identify the soluble target cells within a mixed population.

**Figure 1:**
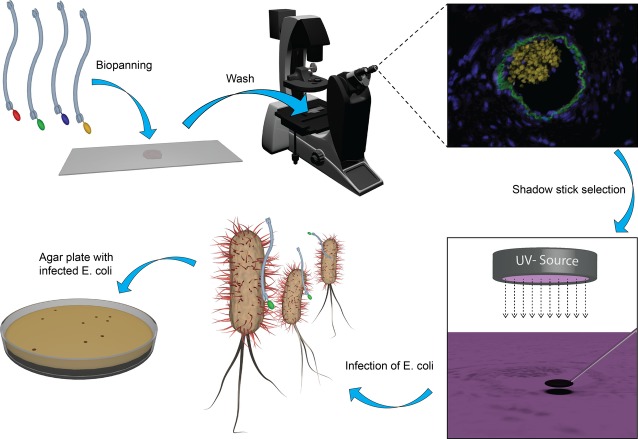
Illustration of the antibody fragment selection procedure by phage display on tissue sections. After target identification the tissue slide is incubated with a phage library. The target area is relocated and a minute disc (shadow stick) is positioned precisely above the cells of interest. The shadow stick shields the phage antibodies binding to the cells of interest from UV-C irradiation. The phages are eluted, but only those protected by the shadow stick may infect bacteria and provide ampicillin resistance. For illustrative purposes, a target area is displayed which is similar to the blood vessel shown in the IHC experiment in Figure6.

**Figure 2:**
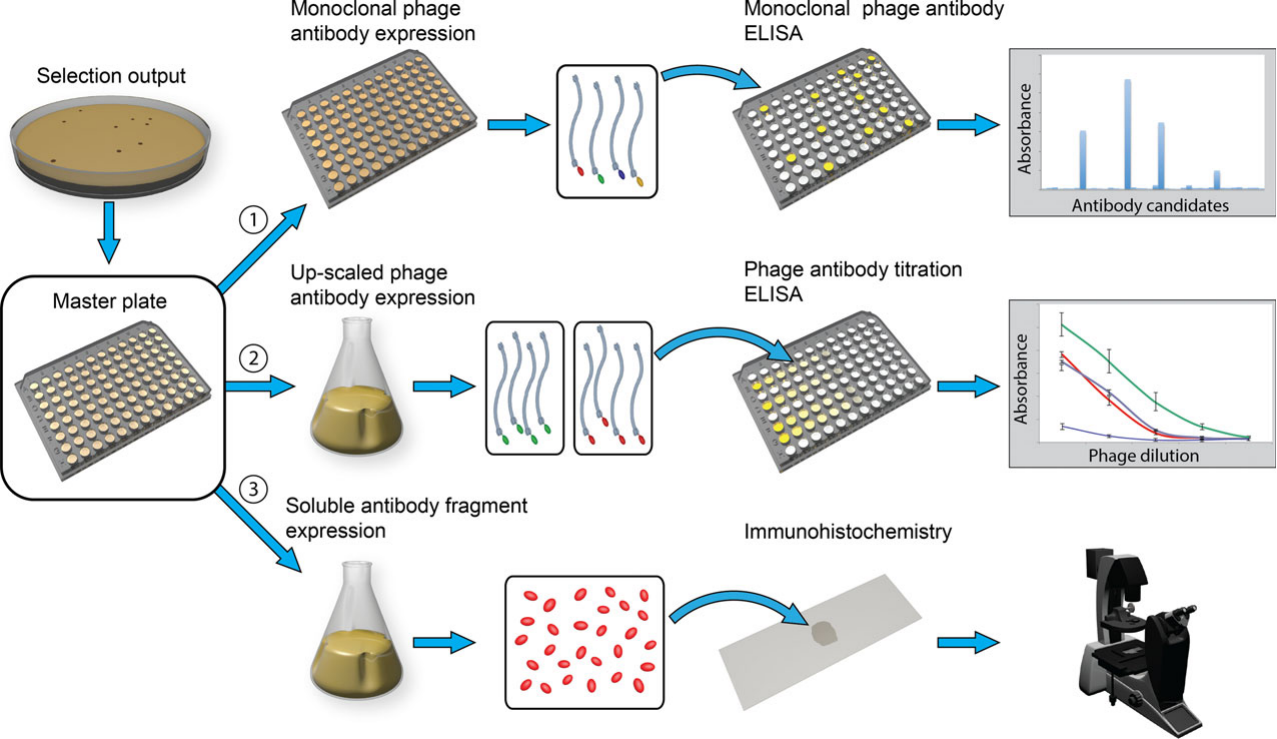
Illustration of the phage antibody screening procedure. Each colony represents a unique antibody fragment which requires screening for its specificity towards the cells of interest. (1) All colonies are grown in microtitre plates and monoclonal phage antibodies are produced. The phage antibodies are initially screened by phage ELISA on cultured endothelial cells. (2) Potentially interesting phage antibodies from the initial screening are monoclonal produced in 50 ml cultures. These are tested in different concentrations by a titration assay which provides comparative results of each phage antibody. (3) Soluble antibody fragments are expressed and purified, and examined by ICC and IHC experiments to validate their specificity.

This study describes a method to select antibody fragments against rare cells by phage display on tissue sections, hereby providing a unique and authentic source for selection as the target niche and microenvironment are preserved. Within a tissue section the target cells or area for antibody fragment selection is identified by either morphology or by a combination of different markers. Biopanning is performed on the entire tissue slide and a minute disc (shadow stick) is positioned precisely above the cells of interest before UV-C irradiation, which render unprotected phages unable to replicate in *E. coli* (Fig.1). Upon propagation in *E. coli* infectious phage antibodies are subsequently screened to identify the cell-specific clones (Fig.2). Finally, soluble antibody fragments are expressed, purified, and tested by immunocytochemistry (ICC) and immunohistochemistry (IHC) experiments to validate their specificity.

## Materials and methods

### Preparation of tissue sections for selection

Formalin fixed and paraffin embedded (FFPE) breast tissue sections from breast reduction surgery of a healthy Danish woman was kindly provided by IN-Lab Medico Aps, Virum, DK. Deparaffinization was performed with HistoChoice Clearing Agent (Sigma-Aldrich, St Louis, MO, USA).

### Target identification by immunohistochemical staining

Antigen retrieval was conducted on the deparaffinized tissue sections with BD Biopharmingen Retreivagen A, according to the manufactures instructions (BD Bioscience, San Jose, CA, USA). The tissue sections were blocked in 4% Marvel dried skimmed milk powder (MPBS) for 1 hr. Then they were incubated with 100 μl of either anti-CD31 mouse monoclonal antibody [P2B1] or rabbit polyclonal von Willebrand Factor (vWF) antibody (Abcam, Cambridge, UK) 1:500 in 2% MPBS under cover glass for 1 hr. The slides were washed two times in PBS and further incubated with 100 μl secondary antibody, Alexa Fluor 488 conjugated Goat Anti-mouse IgG or Anti-rabbit IgG (Invitrogen, Carlsbad, CA, USA) 1:40 in 2% MPBS for 1 hr under cover glass. The slides were washed three times in PBS before being mounted with Vectashield inc. DAPI (Vector Laboratories, Burlingame, CA, USA). Visualization was performed with a Leica DMI3000 B Fluorescence microscope (Leica Microsystems, Wetzlar, Germany), and an Olympus DP72 digital camera with Cell^B image acquisition software (Olympus, Tokyo, Japan). A blood vessel harbouring fluorescent positive cells was identified on the tissue, and the location marked underneath the slide with a diamond tip glass cutter pen. This marking allowed for subsequent relocation by brightfield microscopy and proper placement of the shadow stick above target area.

### Shadow stick

A custom made glass stick with a flattened gold disc at the end was manufactured by Unisense, Aarhus, Denmark. The gold disc is attached on the 1 mm glass capillary with an angle of 135°, which allows positioning of the disc on top of the target area using micromanipulation equipment from Narishige (model MM-188; Nikon, Tokyo, Japan). For the present selections on blood vessels, a gold disc with a diameter of approximately 120 μm was used.

### Shadow stick-selection of antibody fragments using phage display

The cover glass was removed from the tissue section slide by soaking 15 min. in PBS. The mounting medium was removed by washing three times in PBS. The slide was blocked 1 hr in 4% MPBS before incubation with a phage library in a slide container containing 20 ml 2% MPBS for 2 hrs with gentle agitation. 100 μl of both the Tomlinson I and Tomlinson J phagemid scFv antibody libraries were used bearing approximately 100 copies of each individual phage antibody (Source Biosciences, Nottingham, UK) 30. The slide was washed three times 15 min. in PBS and 15 min. in PBS with 10% glycerol (PBSG) with gentle agitation. The slide was dried except from the target area, which was kept moist with approximately 10 μl PBSG. Using brightfield microscopy, the shadow stick was by means of micromanipulation equipment positioned over the target area with the CD31 or vWF positive cells in the centre. The slide was exposed to UV-C light (254 nm) for 12 min. using a UV-C source (model UVSL-14P from UVP, Upland, CA, USA) positioned on a stand approximately 5 cm above the slide. Phage particles bound to the target area was eluted with 100 μl trypsin (1 mg/ml) for 10 min. Trypsin was aspirated and transferred to a tube before the area was washed eight times with 50 μl PBSG, which was transferred to the eluate as well. For trypsin inactivation 50 μl foetal bovine serum was added to the eluate before storage at −20°C. This procedure for shadow stick selection on tissue is based on previously described protocols for single cell selection 16,17.

### Phage infection

2 ml of a grown culture of the *E. coli* strain TG-1 was infected at an OD_600_ between 0.4 and 0.5 with the eluate for 45 min. at 37°C. The infected TG-1 culture was spun down and resuspended in approximately 200 μl culture supernatant and plated on TYE plates with 100 μg/ml ampicillin and 1% glucose, diameter 70 mm. The plate was incubated overnight at 30°C. Single colonies were picked in 96-well plates with 2xTY media containing 100 μg/ml ampicillin and 1% glucose. Phage antibodies were then produced, as described in the Tomlinson protocol using the KM13 helper phage 31. Culturing of *E. coli* and recipes for culture media, *etc*. were performed as described in the Tomlinson protocol 32.

### Phage ELISA for screening and titration assay

ELISA plates, Costar 96 well culture plate (Corning, New York, USA), were prepared by seeding approximately 20,000 human dermal microvascular endothelial cells (HMEC-1) per well and culturing them overnight at 37°C, 5% CO_2_. The HMEC-1 cell line is a simian virus 40 large T antigen immortalized human dermal microvascular endothelial cell line 33. The cells were washed two times in PBS and then fixed in 2% PFA for 10 min. followed by 10 min. in ice cold methanol. Free plastic was blocked using 4% MPBS for 1 hr.

For ELISA screening 50 μl supernatant from the monoclonal phage rescue was mixed with 50 μl 2% MPBS and applied to each well. Each phage antibody was screened in duplicate. As a positive control the phage antibody 8H, which is specific for endothelial cells (not published), was included. As a negative control the KM13 helper phage was included. Approximately 10^11^ phages/well of the controls were added and performed in triplicates. For titration assay, phages were produced in 50 ml TG-1 cultures and PEG precipitated according to the Tomlinson protocol. Phage particles were quantified by measuring absorbance at 269 and 320 nm 34. The phage antibodies of interest were tested along with the above controls, in a series of five 10-fold serial dilutions, ranging from 10^11^ phages/well to 10^7^ phages/well. Each phage antibody was tested in quadruplicate.

The plate was washed and bound phage antibodies were detected with HRP conjugated M13 phage antibody (GE healthcare, Wauwatosa, WI, USA) 1:5000 in 2% MPBS. The plate was washed and development performed with 3,3′,5,5′-tetramethylbenzidine (TMB) Plus ‘Ready-to-use’ substrate solution (Kem-En-Tec Diagnostics, Taastrup, Denmark) according to manufacturer’s protocol. Reaction time: 5–15 min. Absorbance (OD_450_-OD_655_) was read on a Bio-Rad Model 550 microplate reader (Bio-Rad, Hercules, CA, USA) at OD_450_ and OD_655_ (background reference). OD_655_ was subtracted from OD_450_ using the Bio-Rad Microplate Manager 5.2 program.

### Expression and purification of soluble antibody fragments

The interesting phage antibodies were used to infect the *E. coli* strain KS1000 and the soluble scFv antibody fragments expressed. The 6xHis-tagged scFvs were purified from cleared sonicated cell lysates by IMAC purification on Ni-NTA Superflow beads (Qiagen, Germantown, MD, USA) under native conditions according to (The QIAexpressionist, 2003) 35. ScFv containing fractions were identified by SDS-PAGE and dialysed in buffer (300 mM NaCl, 50 mM Trizma base, pH 7.5).

### Cells for ICC

The following four cell lines were applied for ICC: HMEC-1 cell as earlier described. Normal adult skin fibroblasts (ASF-2), derived from a breast biopsy from a consenting 28-year old Danish woman 36. Michigan Cancer Foundation cell line 7 (MCF-7), a human breast adenocarcinoma cell line, kindly donated by Institute of Cellular and Molecular Medicine, Copenhagen University, Denmark 37. The human bone marrow-derived mesenchymal cell line hMSC-TERT created by stable retroviral transfected with the catalytic subunit of telomerase with reverse transcriptase activity 38.

### Immunocytochemical staining with scFv antibody fragments

Approximately 60,000 cells of the different cell types were seeded and cultured overnight in 8-well Nunc Lab-Tek chamber slides (Thermo Scientific, Roskilde, Denmark). ScFvs, 50 μg per well, were added directly to the culture medium and incubated 1 hr with gentle agitation. The cells were washed two times in PBS and subsequently fixed with ice cold methanol for 10 min. After washing two times in PBS, scFvs were detected with mouse Cy3 conjugated anti-c-Myc antibody [9E10] (Sigma-Aldrich) 1:100 in 2% MPBS, 150 μl per well. After 1 hr incubation in the dark with gentle agitation, the slides were washed three times in PBS. Chambers were removed and the slides mounted with Vectashield incl. DAPI and cover glass. The stained cells were analysed with fluorescence microscope, as earlier described. As a negative control, the mouse Cy3 conjugated anti-c-Myc antibody was used without incubation with scFvs.

### Immunohistochemical staining with scFv antibody fragments

The (FFPE) breast tissue sections were deparaffinised as described earlier, but the antigens were retrieved by low-temperature heat induced epitope retrieval in TEG-buffer (10 mM Tris, 0.5 mM EGTA, pH 9) for 12 hrs at 60°C with subsequent slow cooling to room temperature. The slide was washed three times in PBS and blocked for 1 hr in 4% MPBS. The area with tissue section was encircled with a PAP pen liquid blocker. 100 μl PBS containing approximately 60 μg scFv was added to this area and incubated for 1 hr with gentle agitation. The liquid was removed by aspiration and the slide washed three times 1 min. in PBS. ScFvs bound to the tissue were detected with 100 μl mouse Cy3 conjugated anti-c-Myc antibody [9E10] (Sigma-Aldrich) 1:50 in 2% MPBS. The slide was incubated 1 hr in the dark with gentle agitation. The slide was washed three times 1 min. in PBS and mounted with Vectashield incl. DAPI and cover glass.

## Results

### Optimizing the method for selection on tissue

This procedure for shadow stick selection on tissue is based on our previously published protocols for selection against single cells in suspension 16,17. The stroma present in tissue sections introduce additional challenges, such as increased non-specific adhesion of phage particles, thus requiring changes to the single cell selection protocol.

UV-C irradiation inactivates the ability of the phages to replicate their DNA in the host bacteria 39. However, the exposure time used for single cell selection did not result in complete inactivation of non-specific bound phages to tissue. In order to evaluate the most efficient exposure time multiple FFPE breast tissue sections were incubated with phage, washed and exposed to UV-C for 0, 2, 5, 7, 10, 12, 15 and 20 min., respectively. The number of colonies formed after infection of *E. coli* with the trypsin-eluted phages was estimated. As expected, the amount of colonies declined drastically as a result of phage inactivation, due to the increasing UV-C exposure (data not shown). A similar experiment was performed with the shadow stick covering an area on the tissue section. Generally, it generated a slightly higher count of colonies at the corresponding durations (data not shown) and even after 20 min. of exposure a small number of phages could be eluted which allowed replication in *E. coli*. This was expected, as the shadow stick shielded a small area of the tissue from UV-C irradiation and hereby prevented phage inactivation in this particular area. In these experiments, the lowest amount of recovered phages was observed after UV-C exposure for 12 min. This duration was hence adopted as the optimal UV-C exposure. Longer durations of exposure proved suboptimal due to evaporation.

### Selections and screening

**Figure 3:**
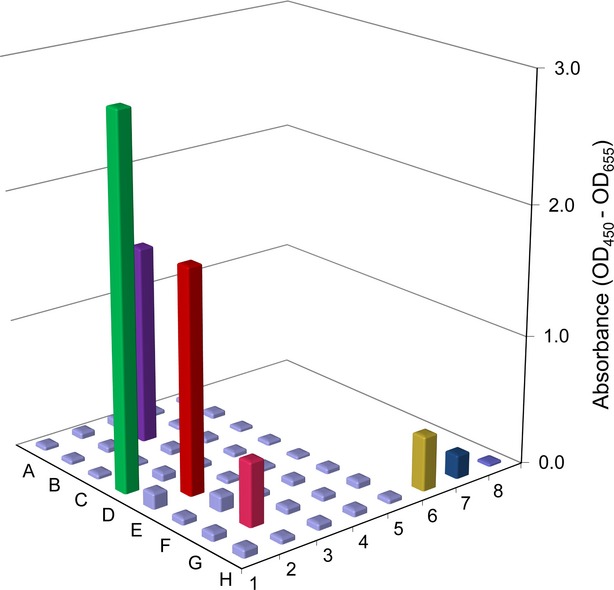
Phage ELISA for screening of the 40 selected phage antibodies. The ELISA was performed in duplicate on HMEC-1 cells of which the average absorbance (OD_450_-OD_655_) is shown. The results are shown in five rows with eight phage antibodies each. Furthest to the right are shown three controls performed in triplicate (row 6–8). The controls are: a confirmed unpublished endothelial cell-specific phage antibody named 8H (row 6), the KM13 helper phage (row 7) (both 10^11^ phages/well), and the secondary antibody alone (HRP-anti-M13) (row 8). The phage antibodies 1D (green), 2E (red), 2G (magenta) and 3B (dark purple) performed well.

Two selections were performed with the Tomlinson library on FFPE breast tissue sections using the shadow stick as described. The target areas contained CD31 or vWF positive cells in blood vessels detected by fluorescence microscopy. The two selections yielded 27 and 13 clones, respectively. These 40 clones were initially screened in duplicate by phage ELISA on the endothelial cell line HMEC-1 with phage antibodies produced in 96-well format (Fig.3). As a positive control, the phage antibody 8H, which is specific for endothelial cells (not published), was included. As negative controls, the KM13 helper phage was included as well as the secondary antibody used alone (HRP-anti-M13). This ELISA screening yielded four phage antibodies: 1D, 2E, 2G and 3B, which showed considerable higher absorbance compared with negative controls. They all derived from the selection on CD31 positive cells. These phage antibodies were further analysed by a titration assay.

### Titration assay

**Figure 4:**
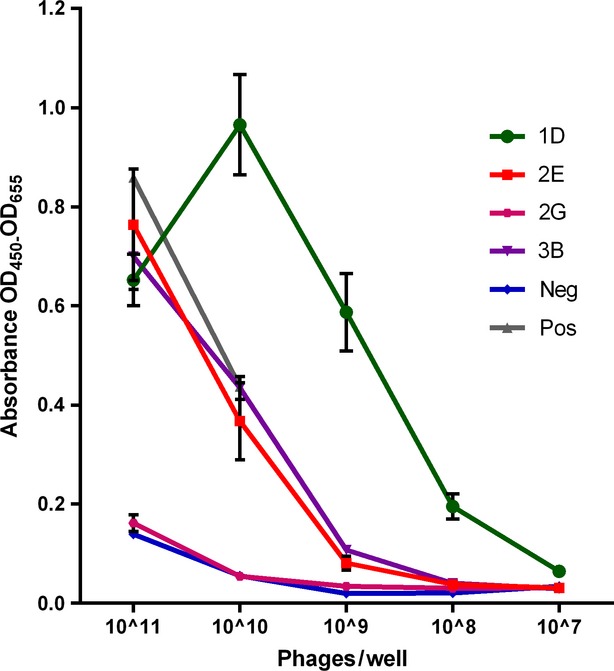
Titration assay performed by phage ELISA with PEG precipitated phage antibodies on HMEC-1 cells. The average absorbance (OD_450_-OD_655_) of the four phage antibodies measured in quadruplicates is shown. *X*-axis indicates phage concentration in number of phages/well. The negative control is the KM13 helper phage (blue). The positive control is the confirmed endothelial cell-specific phage antibody named 8H (grey). Controls are means of duplicate measurements. The phage antibodies 1D (green), 2E (red) and 3B (purple) performed well as opposed to 2G (magenta).

The four phage antibodies were tested by phage ELISA on HMEC-1 cells in a series of five 10-fold serial dilutions, ranging from 10^11^ phages/well to 10^7^ phages/well. Each phage antibody was tested in quadruplicate. As controls, the endothelial cell specific phage antibody 8H and the KM13 helper phage were included. The results show, that the phage antibody 2G does not bind to HMEC-1 cells any better than the negative control (Fig.4). This is in accordance with the screening results, where this phage antibody had the weakest absorbance among the four phage antibodies of interest (Fig.3). The three other phage antibodies showed a high absorbance with 1D, even exceeding the positive control 8H.

### Validation of the antibody fragments by ICC

**Figure 5:**
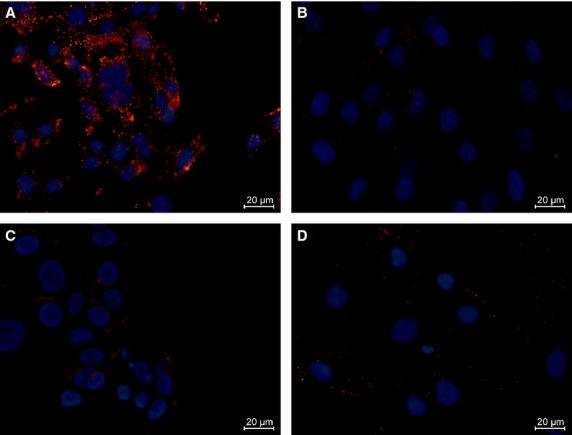
Immunocytochemistry on four cell types. The cell lines are: (A) Immortalised human dermal microvascular endothelial cells (HMEC-1), (B) adult skin fibroblasts (ASF-2), (C) breast adenocarcinoma cells (MCF-7) and (D) immortalized bone marrow-derived mesenchymal cells (hMSC-TERT). The soluble scFv antibody fragment purified from clone 2E shows strong preference for endothelial cells. Cells were co-stained with DAPI and the merged pictures here shown were taken using a 63× objective lens.

Soluble scFv antibody fragments of the three clones 1D, 2E and 3B, were expressed and purified. ICC was performed on four different cell lines: Immortalized HMEC-1, ASF-2, breast adenocarcinoma cells (MCF-7) and immortalized bone marrow-derived mesenchymal cells (hMSC-TERT) 33,36–38. In all the performed ICC experiments, including those performed with the negative control, the MCF-7 cancer cell line displayed a weak consistent staining. This staining is likely attributed to upregulation of the proto-oncogene c-Myc in this particular cell line, and the following binding of the mouse Cy3 conjugated anti-c-Myc antibody used for detection 40,41. 2E displayed strong positive binding to the HMEC-1 cell line. Only minor reactions were found in the other three cell lines (Fig.5). This exclusive staining pattern towards endothelial cells indicates that this particular antibody fragment could be specific towards the same type of cell that it was selected against. 3B did not provide any positive staining in any of the four cell types, despite repeated attempts. 1D showed a weak binding to the HMEC-1 cell line and none in the other three cell lines (data not shown). For both antibodies the presence of full length tag sequences was verified by Western blotting as described in 42 (data not shown). The specificity of the three antibody fragments was further tested by IHC.

### Test of antibody specificity by IHC

**Figure 6:**
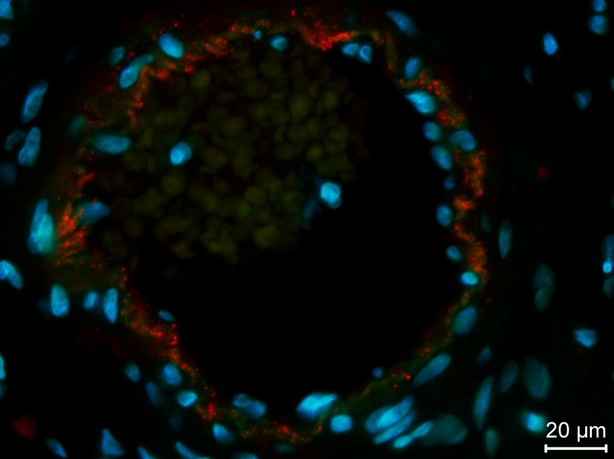
Immunohistochemistry on healthy FFPE breast tissue with the soluble scFv antibody fragment purified from clone 2E. The picture displays an area with a cross-sectioned blood vessel including autofluorescent erythrocytes and vascular lining. The soluble scFv antibody fragment 2E shows strong preference towards the endothelial cells in the vascular lining. The tissue was co-stained with DAPI and pictures taken using a 63× objective lens.

Immunohistochemistry was performed on FFPE breast tissue sections with the three antibody fragments: 1D, 2E and 3B. In all attempts, 2E displayed strong and specific reactions towards the linings of vascular tubes (Fig.6). This is in line with the ICC results, and together these results show that 2E has a strong binding specificity towards endothelial cells. As described earlier, there were no significant reactions with 3B. 1D showed several positive reactions throughout the tissue. However, these were not restricted to endothelial cells, but seemed to include mammary epithelial cells (*not shown*).

## Discussion

The main advantage of the shadow stick technique is the ability to select antibody fragments binding to antigens specifically presented by rare cells mixed within a large heterogeneous population. During biopanning, phage antibodies recognizing antigens shared by many cell types will be depleted by the surrounding tissue. Only a few phage antibodies will bind to the unique or upregulated antigens on the rare cells protected from the UV-C irradiation by the shadow stick. Thus, the shadow stick infers the strength of the selection method *i.e*. providing few and highly relevant output clones.

However, efficient application of the method does have an essential requirement. It is important that the target cells have in fact a low prevalence in the selection material. If targets cells are too abundant, the potentially interesting phage antibodies will be diluted by competitive binding to target cells not present at the minute area covered by the shadow stick. Consequently, those clones will be lost in the selection. When applying the method on rare cells in a suspension, the amount of rare target cells present on the selection slide can easily be adjusted by simple dilution. Such dilution is not possible with tissue. Taking this effect into consideration, tissue samples should be thoroughly evaluated for their potential suitability for the shadow stick method. In the present study, the vascular tubes were naturally scarce. In addition they were cross-sectional and small enough for the shadow stick to provide good coverage limiting loss of potentially interesting phage antibodies. Thus, for the purpose of establishing a proof of concept for the shadow stick selection technique on tissue these sections matched the criteria.

In general, when performing phage antibody selection on cells, protocols often include depletion steps on irrelevant target cells prior to the selection in order to reduce the number of phage antibodies binding to common epitopes 28,29,43. However, when performing depletion, there is an increased risk of removing relevant phage antibodies from the library before the biopanning step on the target cells. It is very likely that the target cells do not present epitopes which are entirely unique for that particular cell, but rather overexpressed. Isolating phage antibodies binding such epitopes could prove highly useful despite that they are not entirely unique. In addition, most phage antibody libraries applied today are in the phagemid format. In such libraries only 1–10% of phage particles present an antibody fragment on their surface, therefore 90–99% of the phages cannot be depleted, however, they still carry antibody genes in their genome and thereby limit the effectiveness of the depletion step. Another strategy may be the use of enrichment steps with multiple rounds of biopanning to enhance the number of relevant binding phage antibodies in the phage library 43–46. However, performing multiple rounds of enrichment favours high-affinity binding antibody fragments as well as antibody fragments binding to antigens present in high concentration on the cells. Moreover, multiple rounds also favour the clones with growth advantages. For example clones with stop codons or truncations in the antibody gene may outcompete the clones of interest 47.

The present method demonstrates an alternative to strategies including depletion steps and/or consecutive biopanning steps. This is due to the fact that the rare target cells are surrounded by a heterogeneous tissue, which serves as a sink for the common epitope binding phage antibodies by simple competitive binding. This depletes the phage antibodies binding to common epitopes on the target cells resulting in a low output of clones, but with a high yield of relevant antibody fragments.

Previously, laser capture microdissection (LCM) has been applied as well in the generation of peptides and antibody fragments binding to specific tissues using phage display 43–45,48,49. However the tissue dehydration, fixation and laser excision following the biopanning step in such procedures, has proven to cause difficulties with eluting viable phage particles using LCM 43–45.

In the present study, two single-round selections were performed on blood vessels with the combined output of 40 clones. Four of the phage antibodies gave relatively high absorbance values in the initial screening by phage ELISA. In the initial phage ELISA, phage particles were produced in 96 well plates, which results in a screening with a variable and generally low concentration of phage antibodies. Therefore, the initial phage ELISA often gives variable results, as the individual phage antibodies saturate the target antigens differently. Additionally, the corresponding target antigens are not represented in equal numbers. Some antigens might be rarely expressed on each cell and give weak signals although being fully saturated with phage antibodies. For the phage antibodies giving a positive signal in the initial phage ELISA, expression were scaled up allowing saturating amounts of phage antibodies to be added and a titration assay performed. Here, three of the phage antibodies showed better binding to endothelial cells compared with 2G and negative control. After purification of soluble scFvs corresponding to these three positive phage antibodies, one of the scFvs proved specific for endothelial cells by both ICC and IHC. This reduction in numbers, from 40 selected to one specific phage antibody, was expected and the reduction can be explained by characterizing the 40 phage antibodies into five groups; One group originated from phage antibodies which, despite the repeated washing steps, bound to the target area unspecifically and was provided with an unintentional protection from the UV-C irradiation by the shadow stick. Another group consisted of phage antibodies that bound specifically to the protected target area, but instead of endothelial cells, they bound to irrelevant neighbouring cells or components in the extracellular matrix and stroma. Even if the size and shape of the shadow stick were customized to perfectly fit a desired target area, some level of undesired protection on the neighbouring cells seem hard to avoid completely. This presents a minor drawback of the use of tissue compared with cells in suspension. As the phage antibodies were screened by phage ELISA on an entirely different cell line, phage antibodies from this group would not necessarily give a reaction on the endothelial cells used.

The phage antibody 2G likely belong to either of the groups described above. This phage antibody gave weak absorbance values in the initial screening by phage ELISA (Fig.3), but further tests in the titration assay revealed that this phage antibody was no better than the negative control (Fig.4). Likely, this difference between the screening phage ELISA and the titration assay can be attributed to unspecific binding and the potential unequal phage concentration occurring from phage production in 96-well format. These problems are diminished in the titration assay as the phage antibody concentrations are equalized before the assay.

The third group of clones originates from phage antibodies which bound to the endothelial target cells, but to a common epitope present in a multitude of different cells. Likely, the clone 1D could belong to this group. 1D gave the highest absorbance values in both the initial screening by phage ELISA and in the titration assay (Figs3 and 4). In the titration assay, a lower absorbance is observed at the concentration of 10^11^ phages/well (Fig.4). This causes an initial increase in absorbance with declining concentration to 10^10^ phages/well. This effect can be explained by some degree of aggregation of phages occurring at high phage concentrations. This effect lowers the ability of phage antibodies to bind to the cells, and causes a lower absorbance at the concentration of 10^11^ phages/well. At lowered concentrations, this problem occurs less and a better binding efficiency is observed. The strong absorbance values observed in both assays could either be attributed to an antigen being present in great abundance in each cell, or be a result of the phage antibody binding with high affinity. However, the following IHC did not show reactions restricted to endothelial cells. Instead, the purified ScFv antibody fragment purified from clone 1D showed promiscuous binding to several different cell types, indicating that this particular antibody fragment binds to a commonly expressed antigen (data not shown). Our initial screening ELISA showed that only 10% of the selected phage antibodies were of potential interest. However, had the screening resulted in a significantly higher percentage of interesting phage antibodies, it would be advantageous to perform the following titration assay on multiple cell lines. This would provide the opportunity to omit the phage antibodies binding common and uninteresting antigens from further testing.

The fourth group is the phage antibodies specifically binding to the target cells. 2E belong to this group, since it showed specific reactions towards endothelial cells in both the ICC and IHC experiments (Figs5 and 6). The antibody fragment expressed from this particular clone was selected without any prior knowledge of the antigens on the target endothelial cells, yet the antibody fragment proves to be specific. As the selection were performed on tissue sections previously stained with anti-CD31 antibody, one concern could be that the specific staining observed in ICC and IHC is derived from recognition of the anti-CD31 antibody. However, this can be excluded as the ELISA performed on cultured endothelial cells (Fig.4) clearly indicates that the antibody bind to endothelial cells which was not pre-stained with the anti-CD31 antibody. The selection of this particular antibody fragment clearly demonstrates the successful appliance of the shadow stick selection method on tissue.

Finally, in a study design such as the present, which includes a screening step on cultured cells by phage ELISA, in theory a fifth group of clones exists. This fifth group consists of phage antibodies which bind specifically to the target blood vessels, but do not give a reaction in the phage ELISA on cultured endothelial cells, due to the potential differences in antigen expression between the cells in the target niche on the tissue and the immortalized cultured endothelial cell line. Thus, this screening step on cultured cells potentially negates one of the advantages of using tissue as the cell line does not provide an authentic picture of the target. The screening might hence render a portion of potentially interesting phage antibodies hidden from discovery. In an ideal study design, the screening step by phage ELISA is to be omitted, and instead ScFv antibody fragments should be purified from all selected clones and tested by IHC. Such an approach may even be a necessity, in the cases where selection has been performed on rare target cells to which an equivalent and suitable cell line does not exist. However, it would prove low-throughput and highly labour intensive, in particular in cases where selections have provided a relatively high yield of clones. In the present study, the initial screening of the 40 phage antibodies by phage ELISA on cultured endothelial cells was of great strategic importance. It provided valuable preliminary indications of a select few phage antibodies of potential interest and allowed rapid testing of those clones. The following evaluation by ICC and IHC of antibody fragment 2E confirm that the shadow stick selection method can be applied for use on tissue. What is more, it shows the potential of this method as a tool for discovering novel biomarkers specific to rare cells.
